# Supplementary material for: Poor treatment outcome and associated risk factors among patients with isoniazid mono-resistant tuberculosis: A systematic review and meta-analysis
Source: PLoS One. 2023 Jul 19;18(7):e0286194. doi: 10.1371/journal.pone.0286194 (PMC10355410; doi:10.1371/journal.pone.0286194)
Supplement: S2 Table — (DOCX) [file pone.0286194.s002.docx]

**Search engine**

**PubMed**

| Search number | Query | Sort By | Filters | Search Details | Results | Time |
| --- | --- | --- | --- | --- | --- | --- |
| 5 | ((("Treatment Outcome"[Mesh]) OR (Poor treatment outcome)) OR (treatment outcome)) AND (Isoniazid mono-resistant) | | | ("Treatment Outcome"[MeSH Terms] OR (("poverty"[MeSH Terms] OR "poverty"[All Fields] OR "poor"[All Fields]) AND ("Treatment Outcome"[MeSH Terms] OR ("treatment"[All Fields] AND "outcome"[All Fields]) OR "Treatment Outcome"[All Fields])) OR ("Treatment Outcome"[MeSH Terms] OR ("treatment"[All Fields] AND "outcome"[All Fields]) OR "Treatment Outcome"[All Fields])) AND (("isoniazid"[MeSH Terms] OR "isoniazid"[All Fields] OR "isoniazide"[All Fields]) AND "mono-resistant"[All Fields]) | 18 | 5:26:21 |
| 4 | Isoniazid mono-resistant | | | ("isoniazid"[MeSH Terms] OR "isoniazid"[All Fields] OR "isoniazide"[All Fields]) AND "mono-resistant"[All Fields] | 94 | 5:25:54 |
| 3 | treatment outcome | |  | "treatment outcome"[MeSH Terms] OR ("treatment"[All Fields] AND "outcome"[All Fields]) OR "treatment outcome"[All Fields] | 1,541,879 | 5:25:36 |
| 2 | Poor treatment outcome | | | ("poverty"[MeSH Terms] OR "poverty"[All Fields] OR "poor"[All Fields]) AND ("treatment outcome"[MeSH Terms] OR ("treatment"[All Fields] AND "outcome"[All Fields]) OR "treatment outcome"[All Fields]) | 103,220 | 5:25:11 |
| 1 | "Treatment Outcome"[Mesh] | Most Recent | | "Treatment Outcome"[MeSH Terms] | 1,213,319 | 5:24:48 |
