# Supplementary material for: Poor treatment outcome and associated risk factors among patients with isoniazid mono-resistant tuberculosis: A systematic review and meta-analysis
Source: PLoS One. 2023 Jul 19;18(7):e0286194. doi: 10.1371/journal.pone.0286194 (PMC10355410; doi:10.1371/journal.pone.0286194)
Supplement: S3 Table — (DOCX) [file pone.0286194.s003.docx]

**Quality assessment for the included studies in meta-analysis**

Critical appraisal for cross-sectional/record review studies

| Author, Year | Q1 | | | | Q2 | | | | Q3 | | | | | Q4 | | | | Q5 | | | | Q6 | | | | Q7 | | | | Q8 | | | | Q9 | | | | Quality score |
| --- | --- | --- | --- | --- | --- | --- | --- | --- | --- | --- | --- | --- | --- | --- | --- | --- | --- | --- | --- | --- | --- | --- | --- | --- | --- | --- | --- | --- | --- | --- | --- | --- | --- | --- | --- | --- | --- | --- |
|  | Y | N | U | NA | Y | N | U | NA | Y | N | U | NA | Y | | N | U | NA | Y | N | U | NA | Y | N | U | NA | Y | N | U | NA | Y | N | U | NA | Y | N | U | NA |  |
| Kwak et al., 2020 | √ |  |  |  | √ |  |  |  | √ |  |  |  |  | | √ |  |  | √ |  |  |  | √ |  |  |  | √ |  |  |  | √ |  |  |  | √ |  |  |  | High |
| Binkhamis et al., 2021 | √ |  |  |  | √ |  |  |  |  | √ |  |  |  | |  |  |  | √ |  |  |  | √ |  |  |  | √ |  |  |  | √ |  |  |  | √ |  |  |  | High |
| Garcia et al., 2018 | √ |  |  |  | √ |  |  |  | √ |  |  |  |  | | √ |  |  | √ |  |  |  | √ |  |  |  | √ |  |  |  | √ |  |  |  | √ |  |  |  | High |
| Karo et al., 2018 | √ |  |  |  | √ |  |  |  | √ |  |  |  |  | |  |  |  | √ |  |  |  | √ |  |  |  | √ |  |  |  | √ |  |  |  | √ |  |  |  | High |
| Romanowski et al., 2017 | √ |  |  |  | √ |  |  |  | √ |  |  |  |  | |  |  |  | √ |  |  |  | √ |  |  |  | √ |  |  |  | √ |  |  |  | √ |  |  |  | High |
| Santos et al., 2018 | √ |  |  |  | √ |  |  |  | √ |  |  |  |  | |  |  |  | √ |  |  |  | √ |  |  |  | √ |  |  |  | √ |  |  |  | √ |  |  |  | High |
| Kuaban et al., 2020 | √ |  |  |  | √ |  |  |  |  | √ |  |  |  | |  |  |  | √ |  |  |  | √ |  |  |  | √ |  |  |  | √ |  |  |  | √ |  |  |  | High |
| Nagar et al., 2022 | √ |  |  |  | √ |  |  |  | √ |  |  |  |  | | √ |  |  | √ |  |  |  | √ |  |  |  | √ |  |  |  | √ |  |  |  | √ |  |  |  | High |
| Chunrong et al., 2020 | √ |  |  |  | √ |  |  |  | √ |  |  |  |  | | √ |  |  | √ |  |  |  | √ |  |  |  | √ |  |  |  | √ |  |  |  | √ |  |  |  | High |
| Garg et al., 2019 | √ |  |  |  | √ |  |  |  |  | √ |  |  |  | |  |  |  | √ |  |  |  | √ |  |  |  | √ |  |  |  | √ |  |  |  | √ |  |  |  | High |

***Y=yes, N=no, U=unclear, NA=not applicable, <60%=low,60-80%=medium, >80%=high quality***

Critical appraisal for case control studies

| Author, year | Q1 | | | | Q2 | | | | Q3 | | | | | Q4 | | | | Q5 | | | | Q6 | | | | | Q7 | | | | | Q8 | | | | | Q9 | | | | | Q10 | | | | Quality score |
| --- | --- | --- | --- | --- | --- | --- | --- | --- | --- | --- | --- | --- | --- | --- | --- | --- | --- | --- | --- | --- | --- | --- | --- | --- | --- | --- | --- | --- | --- | --- | --- | --- | --- | --- | --- | --- | --- | --- | --- | --- | --- | --- | --- | --- | --- | --- |
|  | Y | N | U | NA | Y | N | U | NA | Y | N | U | NA | Y | | N | U | NA | Y | N | U | NA | Y | N | U | NA | Y | | N | U | NA | Y | | N | U | NA | Y | | N | U | NA | Y | | N | U | NA |  |
| Bachir et al., 2021 | √ |  |  |  | √ |  |  |  | √ |  |  |  | √ | |  |  |  | √ |  |  |  | √ |  |  |  | √ | |  |  |  | √ | |  |  |  | √ | |  |  |  | √ | |  |  |  | High |

****Y=yes, N=no, U=unclear, NA=not applicable, <60%=low,60-80%=medium, >80%=high quality***

| Author, Year | Q1 | | | | Q2 | | | | Q3 | | | | | Q4 | | | | | Q5 | | | | | Q6 | | | | | Q7 | | | | | Q8 | | | | | Q9 | | | | | Q10 | | | | | Q11 | | | | Quality score |
| --- | --- | --- | --- | --- | --- | --- | --- | --- | --- | --- | --- | --- | --- | --- | --- | --- | --- | --- | --- | --- | --- | --- | --- | --- | --- | --- | --- | --- | --- | --- | --- | --- | --- | --- | --- | --- | --- | --- | --- | --- | --- | --- | --- | --- | --- | --- | --- | --- | --- | --- | --- | --- | --- |
|  | Y | N | U | NA | Y | N | U | NA | | Y | N | U | NA | Y | N | U | NA | Y | | N | U | NA | Y | | N | U | NA | Y | | N | U | NA | Y | | N | U | NA | Y | | N | U | NA | Y | | N | U | NA | Y | | N | U | NA |  |
| Chien et al., 2014 | √ |  |  |  | √ |  |  |  | | √ |  |  |  | √ |  |  |  | √ | |  |  |  | √ | |  |  |  | √ | |  |  |  | √ | |  |  |  | √ | |  |  |  | √ | |  |  |  | √ | |  |  |  | High |
| Cattamanchi et al., 2009 | √ |  |  |  | √ |  |  |  | | √ |  |  |  | √ |  |  |  | √ | |  |  |  | √ | |  |  |  | √ | |  |  |  | √ | |  |  |  | √ | |  |  |  | √ | |  |  |  | √ | |  |  |  | High |
| Murwira, et al., 2020 | √ |  |  |  | √ |  |  |  | | √ |  |  |  | √ |  |  |  | √ | |  |  |  | √ | |  |  |  | √ | |  |  |  | √ | |  |  |  | √ | |  |  |  | √ | |  |  |  | √ | |  |  |  | High |
| Chierakul et al., 2014 | √ |  |  |  | √ |  |  |  | | √ |  |  |  |  | √ |  |  |  | | √ |  |  | √ | |  |  |  | √ | |  |  |  | √ | |  |  |  | √ | |  |  |  | √ | |  |  |  | √ | |  |  |  | High |
| Jacobson et al., 2011 | √ |  |  |  | √ |  |  |  | | √ |  |  |  | √ |  |  |  | √ | |  |  |  | √ | |  |  |  | √ | |  |  |  | √ | |  |  |  | √ | |  |  |  | √ | |  |  |  | √ | |  |  |  | High |
| Saldaña et al., 2016 | √ |  |  |  | √ |  |  |  | | √ |  |  |  | √ |  |  |  | √ | |  |  |  | √ | |  |  |  | √ | |  |  |  | √ | |  |  |  | √ | |  |  |  | √ | |  |  |  | √ | |  |  |  | High |
| Villegas et al., 2016 | √ |  |  |  | √ |  |  |  | | √ |  |  |  | √ |  |  |  | √ | |  |  |  | √ | |  |  |  | √ | |  |  |  | √ | |  |  |  | √ | |  |  |  | √ | |  |  |  | √ | |  |  |  | High |
| Edwards et al., 2020 | √ |  |  |  | √ |  |  |  | | √ |  |  |  | √ |  |  |  | √ | |  |  |  | √ | |  |  |  | √ | |  |  |  | √ | |  |  |  | √ | |  |  |  | √ | |  |  |  | √ | |  |  |  | High |
| Wang et al., 2014 | √ |  |  |  | √ |  |  |  | | √ |  |  |  | √ |  |  |  | √ | |  |  |  | √ | |  |  |  | √ | |  |  |  | √ | |  |  |  | √ | |  |  |  | √ | |  |  |  | √ | |  |  |  | High |
| Sayfutdinov et al., 2021 | √ |  |  |  | √ |  |  |  | | √ |  |  |  |  | √ |  |  |  | | √ |  |  | √ | |  |  |  | √ | |  |  |  | √ | |  |  |  | √ | |  |  |  | √ | |  |  |  | √ | |  |  |  | High |
| der Heijden et al., 2017 | √ |  |  |  | √ |  |  |  | | √ |  |  |  |  |  |  | √ |  | |  |  | √ | √ | |  |  |  | √ | |  |  |  | √ | |  |  |  | √ | |  |  |  | √ | |  |  |  | √ | |  |  |  | High |
| Shao et al., 2020 | √ |  |  |  | √ |  |  |  | | √ |  |  |  | √ |  |  |  | √ | |  |  |  | √ | |  |  |  | √ | |  |  |  | √ | |  |  |  | √ | |  |  |  | √ | |  |  |  | √ | |  |  |  | High |
| Salindri et al., 2018 | √ |  |  |  | √ |  |  |  | | √ |  |  |  | √ |  |  |  | √ | |  |  |  | √ | |  |  |  | √ | |  |  |  | √ | |  |  |  | √ | |  |  |  | √ | |  |  |  | √ | |  |  |  | High |
| Tabarsi et al., 2009 | √ |  |  |  | √ |  |  |  | | √ |  |  |  |  | √ |  |  |  | | √ |  |  | √ | |  |  |  | √ | |  |  |  | √ | |  |  |  | √ | |  |  |  | √ | |  |  |  | √ | |  |  |  | High |

Critical appraisal for cohort studies

***Y=yes, N=no, U=unclear, NA=not applicable, <60%=low,60-80%=medium, >80%=high quality***
