# Supplementary material for: Poor treatment outcome and associated risk factors among patients with isoniazid mono-resistant tuberculosis: A systematic review and meta-analysis
Source: PLoS One. 2023 Jul 19;18(7):e0286194. doi: 10.1371/journal.pone.0286194 (PMC10355410; doi:10.1371/journal.pone.0286194)
Supplement: S9 Fig — (DOCX) [file pone.0286194.s012.docx]

**S9 fig.**
