# Supplementary material for: Poor treatment outcome and associated risk factors among patients with isoniazid mono-resistant tuberculosis: A systematic review and meta-analysis
Source: PLoS One. 2023 Jul 19;18(7):e0286194. doi: 10.1371/journal.pone.0286194 (PMC10355410; doi:10.1371/journal.pone.0286194)
Supplement: Attachment — Submitted filename: Response to reviewers.docx [file pone.0286194.s032.docx]

**Revisions based on the Editor’s and the reviewers’ comments and suggestions**

**Title:** Poor treatment outcome and associated risk factors among patients with isoniazid mono-resistant tuberculosis: a systematic review and meta-analysis (PONE-D-22-32583R1)

**Journal Requirements:**

Please review your reference list to ensure that it is complete and correct. If you have cited papers that have been retracted, please include the rationale for doing so in the manuscript text, or remove these references and replace them with relevant current references. Any changes to the reference list should be mentioned in the rebuttal letter that accompanies your revised manuscript. If you need to cite a retracted article, indicate the article’s retracted status in the References list and also include a citation and full reference for the retraction notice.

- **Response: Thank you, the reference list is complete and correct. A retracted paper is not cited.**

**Reviewers' comments:**

Reviewer's Responses to Questions

**Comments to the Author**

1. If the authors have adequately addressed your comments raised in a previous round of review and you feel that this manuscript is now acceptable for publication, you may indicate that here to bypass the “Comments to the Author” section, enter your conflict of interest statement in the “Confidential to Editor” section, and submit your "Accept" recommendation.

Reviewer #1: All comments have been addressed

Reviewer #2: (No Response)

- **Response: Thank you for the pertinent review.**

2. Is the manuscript technically sound, and do the data support the conclusions?

The manuscript must describe a technically sound piece of scientific research with data that supports the conclusions. Experiments must have been conducted rigorously, with appropriate controls, replication, and sample sizes. The conclusions must be drawn appropriately based on the data presented.

Reviewer #1: Yes

Reviewer #2: (No Response)

- **Response: Thank you for the pertinent review.**

3. Has the statistical analysis been performed appropriately and rigorously?

Reviewer #1: Yes

Reviewer #2: (No Response)

- **Response: Thank you for the pertinent review.**

4. Have the authors made all data underlying the findings in their manuscript fully available?

The [PLOS Data policy](http://www.plosone.org/static/policies.action#sharing) requires authors to make all data underlying the findings described in their manuscript fully available without restriction, with rare exception (please refer to the Data Availability Statement in the manuscript PDF file). The data should be provided as part of the manuscript or its supporting information, or deposited to a public repository. For example, in addition to summary statistics, the data points behind means, medians and variance measures should be available. If there are restrictions on publicly sharing data—e.g. participant privacy or use of data from a third party—those must be specified.

Reviewer #1: Yes

Reviewer #2: (No Response)

- **Response: Thank you for the pertinent review.**

5. Is the manuscript presented in an intelligible fashion and written in standard English?

PLOS ONE does not copyedit accepted manuscripts, so the language in submitted articles must be clear, correct, and unambiguous. Any typographical or grammatical errors should be corrected at revision, so please note any specific errors here.

Reviewer #1: Yes

Reviewer #2: (No Response)

- **Response: Thank you for the pertinent review.**

6. Review Comments to the Author

Please use the space provided to explain your answers to the questions above. You may also include additional comments for the author, including concerns about dual publication, research ethics, or publication ethics. (Please upload your review as an attachment if it exceeds 20,000 characters)

**Reviewer #1:**

1. Line 270: please write ….. isoniazid (INH), streptomycin(STR), fluoroquinolones(FLQ), second-line injectable drugs (SLIDs) and continuation phase(rifampicin(RIF), pyrazinamide (PZA)).

- **Response: Thank you for the comment. It is revised accordingly.**

2. Table 1 : Its need you explain in footnote of table1
Cattamanchi et al., 2009*
* the study only indicated the treatment completion rate the total successful treatment outcome including the cured cases and the poor treatment outcome (failure, death and lost to follow-up) are not indicated in the study.

- **Response: Thank you for the valuable comment and suggestion. We revised it based on the given suggestion.**

3.Table 1 : Chien et al., 2014 : All (Median age was 64 years) . What does "all" mean? All age groups? 64 years seems to be high for the middle age, please recheck. For all studies that include all age groups, please add the word "all age groups". For example Binkhamis et al., 202…. all age groups ( range: 1-90 years).

- **Response: Thank you for the valuable comments. We replaced the word “All” with the phrase “all age groups”. The median age described in the Chien et al., 2014 study is 64.0 years as indicated in page number 61 of their study.**

4.(Median zge was 41 years)……. Median age was 41 years

- **Response: Thank you, we revised it.**

Reviewer #2: (No Response

Kind regards,

Ayinalem Alemu

Ethiopian Public Health Institute

National Tuberculosis Reference Laboratory,

P.O. Box 1242, Addis Ababa, Ethiopia.

Email: ayinalemal@gmail.com

Tell: +251912366676
